# Supplementary material for: Nutrient Deficiency Promotes the Entry of Helicobacter pylori Cells into Candida Yeast Cells
Source: Biology (Basel). 2021 May 12;10(5):426. doi: 10.3390/biology10050426 (PMC8151769; doi:10.3390/biology10050426)
Supplement: Supplementary file 1 [file biology-10-00426-s001.zip › biology-1183089-supplementary.pdf]

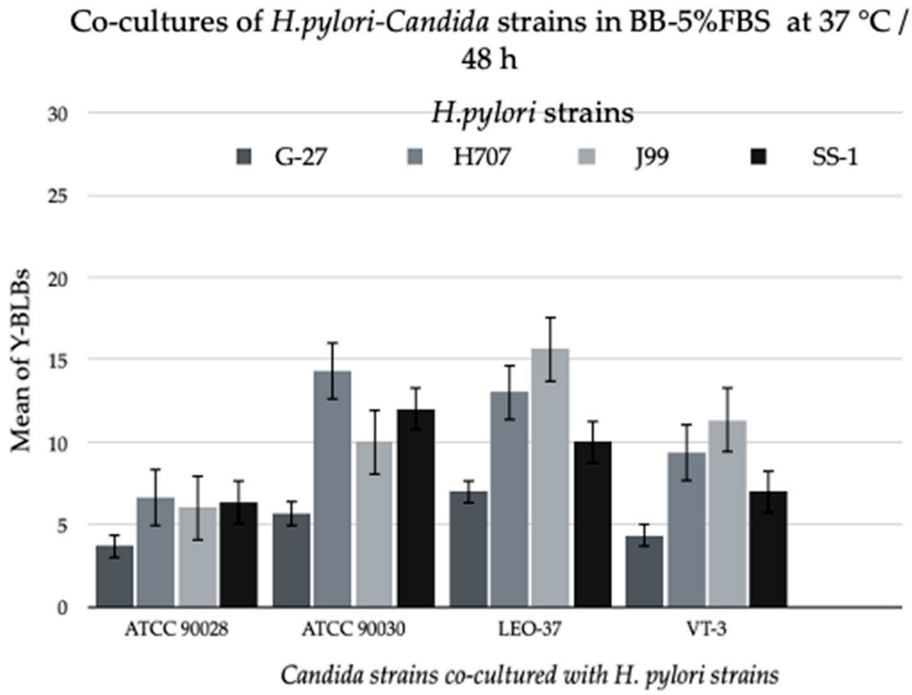

Supplementary Figure S1. Means of yeasts cells harboring bacteria like bodies (Y-BLBS) obtained in 48 h co-cultures incubated in Brucella broth supplemented with 5% fetal bovine serum (BB-5%FBS). The higher means of Y-BLBS were found in co-cultures prepared with *Candida* ATCC 90030, LEO-37 and VT-3 strains.

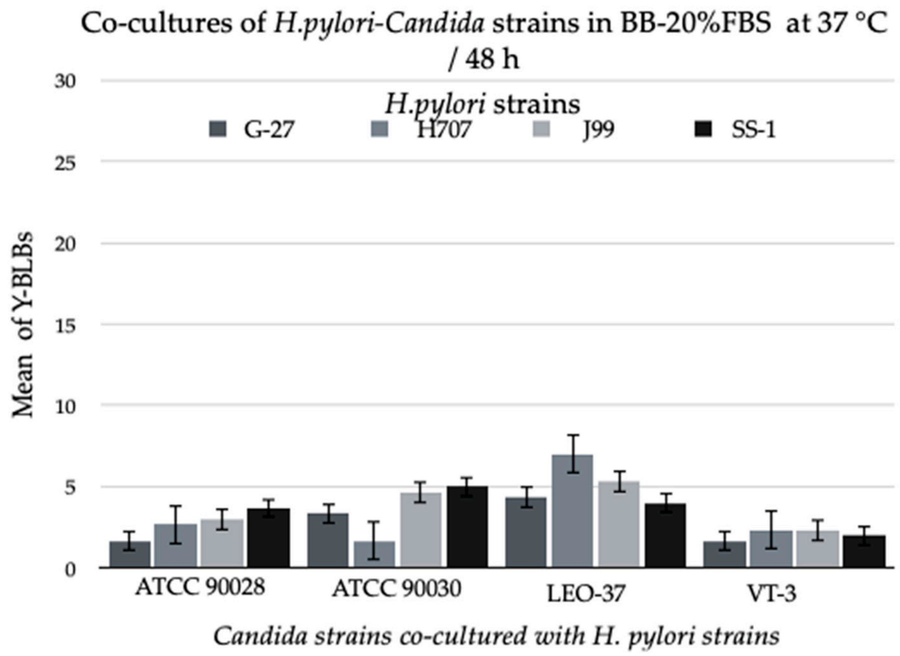

Supplementary Figure S2. Means of yeasts cells harboring bacteria like bodies (Y-BLBS) obtained in 48 h co-cultures incubated in Brucella broth supplemented with 20% fetal bovine serum (BB-20%FBS). The higher means of Y-BLBS were found in co-cultures prepared with *Candida* ATCC 90030 and LEO-37 strains.

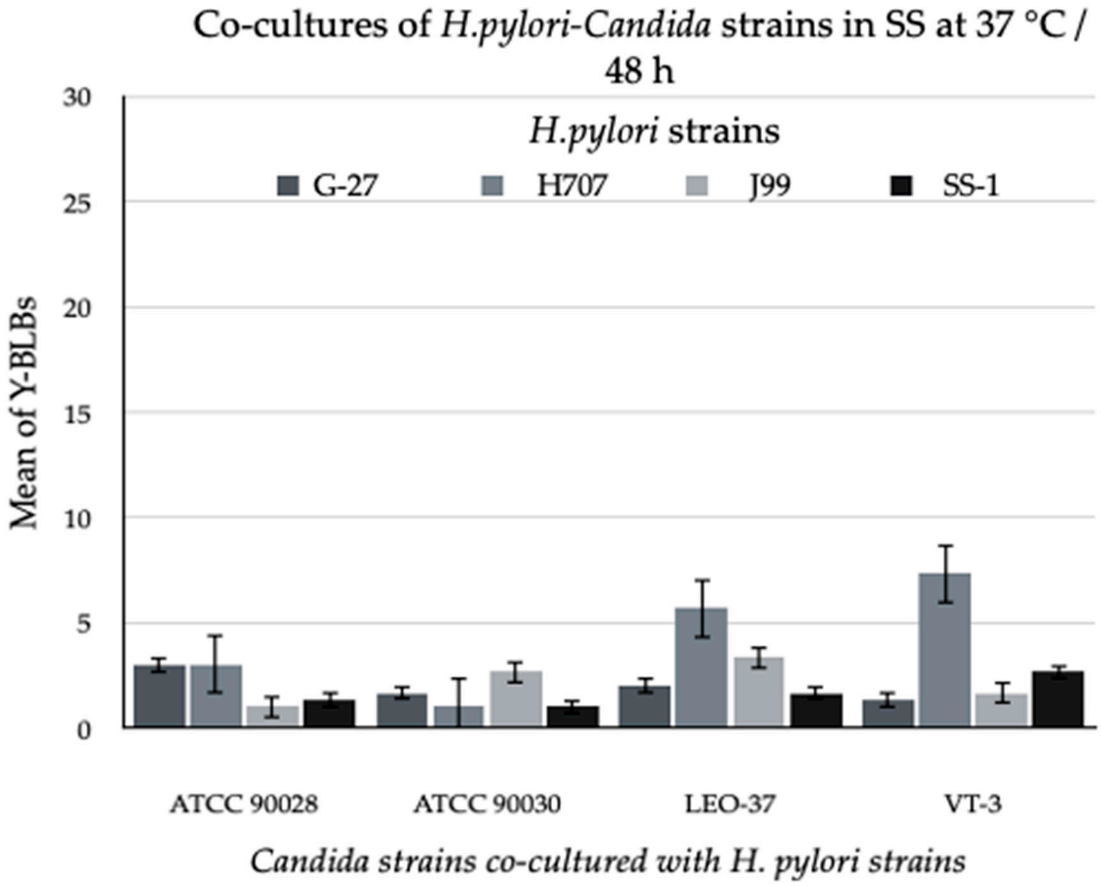

Supplementary Figure S3. Means of yeasts cells harboring bacteria like bodies (Y-BLBS) obtained in 48 h co-cultures incubated in saline solution (SS). The higher means of Y-BLBS were found in co-cultures prepared with *Candida*, LEO-37 and VT-3 strains.

Video S1: Wet mount observed using an optical microscope, of a *H. pylori-Candida* co-culture showing mobile bacteria like bodies within the vacuole of yeast cells. https://youtu.be/CFGR_iproVk
